# Satisfaction Factors with a Dental Unit Chair System in South Korea: A Dentist’s Perspective

**DOI:** 10.3390/healthcare10030437

**Published:** 2022-02-25

**Authors:** Keunbada Son, Young-Tak Son, Myoung-Uk Jin, Kyu-Bok Lee

**Affiliations:** 1 Advanced Dental Device Development Institute, Kyungpook National University, Daegu 41940, Korea; sonkeunbada@gmail.com (K.S.); dudxkr741@naver.com (Y.-T.S.); 2 Department of Dental Science, Graduate School, Kyungpook National University, Daegu 41940, Korea; 3 Department of Conservative Dentistry, School of Dentistry, Kyungpook National University, Daegu 41940, Korea; 4 Department of Prosthodontics, School of Dentistry, Kyungpook National University, Daegu 41940, Korea

**Keywords:** dental unit chair, satisfaction factor, design, dentist, survey

## Abstract

This study aimed to survey users’ satisfaction with a dental unit chair in order to highlight the elements affecting the dentist’s satisfaction. The questionnaire items were drawn up with seven components that constitute a dental unit chair, including the light, patient seat, foot controller, water fountain and cuspidor, monitor, bracket table and controller, and dentist chair. With these questionnaire elements, a pilot experiment was conducted to test the reliability, and reliability analysis was conducted. The scale reliability was checked using Cronbach’s alpha coefficient. Bartlett’s test of sphericity, the Kaiser-Meyer-Olkin (KMO) measure, and factor analysis were performed to test whether the items would constitute appropriate questionnaire items for the survey. The survey was conducted with 26 dentists with more than three years of clinical experience. A correlation analysis was conducted using Pearson’s correlation coefficient (PCC) (α = 0.05) to analyze the impact of the factors on the overall satisfaction with the dental unit chair. The items that were strongly correlated with the overall satisfaction score of the dental unit chair were the design and appearance quality of the dental unit chair (PCC = 0.781), its maintenance (PCC = 0.784), and the overall satisfaction with the water fountain and cuspidor (PCC = 0.703) (*p* < 0.05). Most of the questionnaire items could affect the overall satisfaction with the dental unit chair. Additionally, because the design and appearance quality, maintenance, and overall satisfaction with the water fountain and cuspidor may have the greatest impact on the overall satisfaction with the dental unit chair, the improvement of these elements may bring about the enhancement of the overall satisfaction.

## 1. Introduction

**Figure 1:**
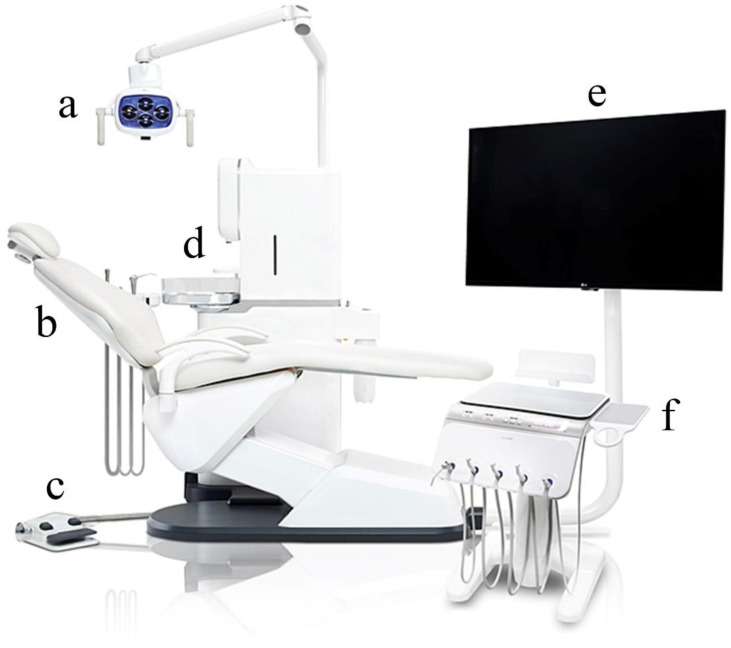
Dental unit chair system. (**a**) Light, (**b**) patient seat, (**c**) foot controller, (**d**) water fountain and cuspidor, (**e**) monitor, and (**f**) bracket table and controller.

In dental clinics, the use of a dental unit chair is essential for the diagnosis and treatment of patients [1,2,3]. Dentists spend most of their office hours in the dental unit chair space for patient care [4,5]. In addition to dentists, patients and dental hygienists also use the dental unit chair [6]. The dental unit chair consists of the following components: the light, patient seat, foot controller, water fountain and cuspidor, monitor, bracket table and controller, and dentist chair (Figure 1).

Regarding the light of the dental unit chair, precise light irradiation should be possible to the patient’s mouth for medical treatment, and convenience of movement is required for the operator [7]. Furthermore, the color temperature control should be convenient, considering tooth color detection and eye fatigue in various clinical environments [8]. Additionally, considering the influence of illumination, illumination suitable for medical treatment is required for resin polymerization and the prevention of eye fatigue [9].

The patient seat of the dental unit chair requires a seat that has an appropriate size for the human body of each country, taking into consideration the body shape of the patient and dentist [10]. Furthermore, the optimal back reclining angle and headrest reclining angle are needed for the patient’s comfort and the dentist’s treatment posture, and appropriate speed is also required, considering the stability and ease of operation when lying or returning the patient to the original position [10]. Additionally, the thickness of the seat is important, considering the shape of the spine and the height of the knee for the dentist’s correct treatment posture [11]. The auxiliary handle of the patient seat should not disturb the patient’s movement, and instead should but help in this regard. All of these factors may have a significant impact on long-term patient care [12,13].

The foot controller should allow the dentist’s intuitive operation and precise use of the handpiece [14]. Additionally, pedal pressures placing less strain on the ankle are required for long-time use [15].

The water fountain and cuspidor should be convenient when they are operated by the patient and assistant [16]. The height and area of the spit sink should be of a size that can minimize inconvenience in movement. Contaminants should be cleaned automatically, and the ease of cleaning management is needed [17]. Additionally, the cup position, the adequacy of the quantity of the water supply, and the water supply speed are important. Furthermore, water purification and wastewater treatment facilities are essential for a pleasant environment and the prevention of cross infection [18,19,20].

The monitor should be visually comfortable, should provide a great deal of information, and should be of a size which is harmonized with the overall treatment system [21]. An appropriate monitor distance between the dentist and the patient is needed, considering a visually comfortable and moving line. Additionally, the monitor should move stably and smoothly, and should be supported and fixed stably even after the movement without any motion. A legible resolution is needed in the treatment process for the display of radiographs, oral photographs, and texts [22].

The bracket table and controller should be of a suitable size for various appliances to be placed on them without affecting the treatment environment [23]. The bracket table and controller should be of a stable weight, should not have any problems with movement, and should allow the stable and convenient mounting and moving of various appliances, as well as providing stability at fixation. Additionally, in the process of controlling several medical devices, a user interface (UI) for the user experience of dentists and assistants is required [24].

The dentist’s chair may affect the dentist’s musculoskeletal health [25,26,27,28]. The backrest angle and shape should be suited for the dentists’ health and comfort, and the design should not burden the human body, especially with the dentist’s long-term use [29,30,31]. Additionally, smooth chair movement is required in the process of treatment, considering durability and noise.

A dental unit chair is a necessity in dental clinics, but due to the continuous development of the dental medical device market, various dental unit chairs are being developed, making it difficult for dentists to properly select a dental unit chair when purchasing the dental unit chair. The dental unit chair causes eye fatigue due to light [8,9]; should feature an appropriate size and angle of the patient seat [10,11,12,13], and the appropriate usability of the foot controller [14,15]; the water fountain and cuspidor should be convenient [16,17]; and there should be harmony between the monitor and the overall treatment system [21,22], as well as size and stability of bracket table and controller [23,24]. It is selected by various factors, including the overall design [29,30,31]. However, it is difficult for dentists to choose a suitable dental unit chair, considering all factors. There are still insufficient studies providing quantitative information regarding the factors to consider while purchasing the dental unit chair. Thus, this study aimed to conduct a survey of user satisfaction with a dental unit chair, and to highlight the elements affecting dentists’ satisfaction. The null hypothesis was that the questionnaire items in this study have no correlation with the overall satisfaction with the dental unit chair.

## 2. Materials and Methods

### 2.1. Elements of the Survey of Dental Unit Chair User Satisfaction

Based on clinical experts’ advice, important questionnaire items were set up regarding the dental unit chair parts (Table 1). The questionnaire consisted of five items on overall satisfaction (the variety of prices and features, design and appearance quality, maintenance, and overall satisfaction) and the items of each element constituting the dental unit chair (light, patient seat, foot controller, water fountain and cuspidor, monitor, bracket table and controller, and dentist chair) (Table 1). The questionnaire items of each component of the dental unit chair were as follows: 4 items on the light, including the convenience of the light head movement, the convenience of the color temperature control, the influence of illuminance, and overall satisfaction; 7 items on the patient seat, including the appropriate length and area for each part, back reclining angle, headrest recline angle, backrest reclining speed, backrest seat thickness, convenience of the auxiliary handle, and overall satisfaction; 3 items on the foot controller, including the ease of operation, pedal pressure during operation, and overall satisfaction; 7 items on the water fountain and cuspidor, including the ease of operation, height and area of the cuspidor, cup position, water purification and wastewater treatment facilities, water quantity and speed, convenience of cleaning the cuspidor, and overall satisfaction; 6 items on the monitor, including the screen size, distance between monitor and the operator/patient, convenience to move, resolution, arm support, and overall satisfaction; 6 items on the bracket table and controller, including the size, weight, mounting stability, controller UI, stability of the table arm operation, and overall satisfaction; and 4 items on the dentist chair, including the backrest angle and shape, convenience to move, human engineering design, and overall satisfaction) (Table 1). The score of each questionnaire item was evaluated on a Likert scale from 1 to 5 (1: very low satisfaction; 2: low satisfaction; 3: neither high nor low; 4: high satisfaction; 5: very high satisfaction).

A pilot experiment was conducted with an additional 5 participants for a reliability test of the questionnaire elements. Regarding the reliability analysis of the scale, the reliability was checked through Cronbach’s alpha coefficient, using statistical software (SPSS release 25.0, IBM, Chicago, IL, USA) (α = 0.05). Previous literature reported that a Cronbach’s alpha of >0.9 indicates reliability and consistency among the items [32,33,34]. Bartlett’s test of sphericity and the Kaiser-Meyer-Olkin (KMO) measure were calculated, indicating the appropriateness of the variable selection for the KMO value factor analysis (>0.9: excellent, 0.8–0.89: good, 0.7–0.79: suitable, 0.6–0.69: ordinary, 0.5–0.59: unsuitable for use, <0.5: impossible for use in a survey) [35].

Factor analysis was conducted to reduce the variables, remove unnecessary variables, identify variable characteristics, and validate the analysis of the measurement items. Furthermore, communality was calculated, and its value shows how well each variable is described by the extracted factors (the communality value is suitable if they are >0.5) [36].

The results of the reliability analysis of the survey elements showed that there were excellent reliability and consistency among them (Cronbach’s alpha = 0.975). The results of the KMO measure and Bartlett’s test of sphericity revealed that there was excellent appropriateness of the variable selection for the factor analysis (KMO = 0.894; *p* < 0.001). The result of the communality calculation (>0.5) showed how well each variable was described by the extracted factors (Table 2). In the questionnaire, factors in seven of the categories showed the explanatory power of 87.217% of the global dispersion, excellent reliability, and consistency among the items (Table 2). Thus, we tested whether the factors of this questionnaire constituted appropriate questionnaire items for the survey of the satisfaction with the dental unit chair.

### 2.2. Survey of Dental Unit Chair User Satisfaction

This survey was conducted with the approval of the Clinical Trial Ethics Committee of Kyungpook National University Dental Hospital (IRB No. KNUDH-2021-04-04-00). The survey was conducted with 26 dentists after the sufficient description of the satisfaction survey with the dental unit chair. Prior to this study, a pilot experiment was conducted with the same materials and methods as this study but with 5 subjects; based on the results, the a priori sample size calculation was performed, using power analysis software (G*Power v3.1.9.2; Heinrich-Heine-Universität Düsseldorf, Düsseldorf, Germany) (N = 26; correlation ρ H1 = 0.66; actual power = 97.36%; power = 97%; α = 0.05). As for the subject selection, two investigators (K.S. and K.-B.L.) counseled the participants, and those who had active opinions and high interests in the dental unit chair were selected as the subjects. Twenty-six participants were enrolled in the study according to the inclusion and exclusion criteria (Table 3). The participants evaluated the dental unit chair systems used in dental clinics, and the manufacturers of the chair unit systems were as follows: Shinhung (Seoul, Korea), Osstem Implant (Seoul, Korea), Sky Dental (Cheongju, Korea), and Dentium (Seoul, Korea). The survey took about 30 min per person. The information on the 26 participants is as follows—sex: 16 men and 10 women; mean age: 37.3 ± 7.7 years; and mean clinical experience: 9.7 ± 6.3 years.

### 2.3. Statistical Analysis

All of the statistical data analyses were performed using statistical software (SPSS release 25.0, IBM, Chicago, IL, USA) (α = 0.05). First, the normal distribution of the data was investigated through the Shapiro–Wilk test, and the normal distribution was checked. A correlation analysis was conducted by assessing Pearson’s correlation coefficient (PCC) in order to analyze the impact of each factor on the overall satisfaction with the dental unit chair. The correlations were divided into perfect correlation (PCC = −1 or 1), strong correlation (PCC = −0.7 to −0.9 or 0.7 to 0.9), ordinary correlation (PCC = −0.4 to −0.6 or 0.4 to 0.6), and weak correlation (PCC = −0.1 to −0.3 or 0.1 to 0.3) [35].

## 3. Results

### 3.1. Results of the Correlations of the Overall Satisfaction Score of the Dental Unit Chair with the Questionnaire Items

The overall satisfaction score of the dental unit chair was significantly correlated with 38 questionnaire items (*p* < 0.05; Table 4), except for the headrest reclining angle of the patient seat (*p* = 0.07), the ergonomic design of the dentist chair (*p* = 0.076), and the overall satisfaction with the dentist chair (*p* = 0.064). Furthermore, the overall satisfaction score of the dental unit chair was strongly correlated with elements such as the quality of the design and appearance of the dental unit chair (PCC = 0.781), maintenance (PCC = 0.784), and the overall satisfaction with the water fountain and cuspidor (PCC = 0.703) (Table 4). All of the other items were ordinarily correlated with the overall satisfaction score of the dental unit chair score (Table 4).

### 3.2. Results of the Correlations of the Overall Satisfaction Score of Each Component (Light, Patient Seat, Foot Controller, Water Fountain and Cuspidor, Monitor, Bracket Table and Controller, and Dentist Chair) of the Dental Unit Chair with the Questionnaire Items

The overall satisfaction with each component of the dental unit chair was significantly correlated with the questionnaire items, except for the convenience of the movement of the light head (*p* = 0.064) (*p* < 0.05; Table 5).

The overall satisfaction with the light of the dental unit chair was strongly correlated with the convenience of the color temperature control (PCC = 0.866) and the influence of the illumination (PCC = 0.803), and highly correlated with the convenience of the color temperature control (Table 5).

The overall satisfaction with the patient seat of the dental unit chair was strongly correlated with the appropriate length and area for each part (PCC = 0.821), back reclining angle (PCC = 0.727), backrest reclining speed (PCC = 0.700), and convenience of the auxiliary handle (PCC = 0.700), and highly correlated with appropriate length and area for each part (Table 5).

The overall satisfaction with the foot controller of the dental unit chair was strongly correlated with ease of operation (PCC = 0.736) and pedal pressure during operation (PCC = 0.737), and highly correlated with the two elements (Table 5).

The overall satisfaction with the water fountain and cuspidor of the dental unit chair was strongly correlated with the ease of operation (PCC = 0.748), height and area of the cuspidor (PCC = 0.824), cup position (PCC = 0.862), water purification and wastewater treatment facilities (PCC = 0.722), and convenience of cleaning the cuspidor (PCC = 0.774), and highly correlated with the height and area of the cuspidor (Table 5).

The overall satisfaction with the monitor of the dental unit chair was strongly correlated with the monitor size (PCC = 0.871), the distance between the monitor and the operator/patient (PCC = 0.923), its convenience to move (PCC = 0.877), its resolution (PCC = 0.811), and its arm support (PCC = 0.865), and highly correlated with the distance between the monitor and the operator/patient (Table 5).

The overall satisfaction with the bracket table and controller of the dental unit chair was strongly correlated with the bracket table and controller size (PCC = 0.824), weight (PCC = 0.948), mounting stability (PCC = 0.899), controller UI (PCC = 0.836), and stability of the table arm operation (PCC = 0.899), and highly correlated with its weight (Table 5).

The overall satisfaction with the dentist chair of the dental unit chair was strongly correlated with the backrest angle and shape (PCC = 0.768), convenience to move (PCC = 0.780), and human engineering design (PCC = 0.932), and highly correlated with the human engineering design (Table 5).

## 4. Discussion

This in vivo study conducted a survey of the user satisfaction with a dental unit chair in order to highlight the factors affecting Korean dentists’ satisfaction. The null hypothesis was that the questionnaire items in this study would not affect the satisfaction with the dental unit chair; however, the null hypothesis was dismissed (*p* < 0.05; Table 2, Table 3 and Table 4). In this study, a reliability test of the questionnaire items was conducted. This study proved that most of the derived factors might affect the overall satisfaction with the dental unit chair.

To the best of our knowledge, in several previous studies on the dental unit chair, there were no studies providing quantitative information regarding the factors to consider while purchasing a dental unit chair. Thus, an appropriate comparison with previous studies is difficult. However, quantitative information on factors to consider when purchasing a dental unit chair can help the dentist in their decision, as it can confirm what items to consider when choosing a dental unit chair. In addition, this information will be important information for dental unit chair manufacturers, as they can check the design preferred by dentists and the factors that are important to them when developing or improving the chair unit. Furthermore, this study may become a new guideline for the evaluation of the chair unit because it was able to derive important factors by evaluating the dental unit chair.

Previous studies have reported on the musculoskeletal risk factors when dentists use a dental unit chair [29,30,31]. Electromyographic evaluation was performed in order to assess the musculoskeletal risks, and it was reported that elements of the dental unit chair design had an effect on the dentist’s musculoskeletal health [27,29,30]. Furthermore, elements of the dental unit chair design may have a significant impact on the aesthetic elements of its appearance. However, they should not affect the dentist’s musculoskeletal health while using the unit for a long time. The design elements of a dental unit chair may possess some risk factors, such as percutaneous injuries [23], the risk of cross infection [17], work convenience [1,2,3], and musculoskeletal diseases. Thus, this study highlighted the design elements that affect the dental unit chair, with two investigators (K.S. and K.-B.L.) subdividing each component of the dental unit chair. Additionally, in this study, a reliability test of the derived elements was performed, and the appropriate reliability for a survey of satisfaction with the dental unit chair was tested.

This study reported that the design and appearance quality, maintenance, and overall satisfaction with the water fountain and cuspidor may have the greatest impact on the overall satisfaction with the dental unit chair (Table 2). From the perspective of dentists, for the overall satisfaction with the dental unit chair, the design and appearance quality of the dental unit chair—which can be checked by the naked eye—would be the most important factors. Additionally, in the interviews after the survey in this study, it was found that the respondents preferred the dental unit chair that could be used stably without any failure for a long time, as they had to receive patients continuously, and placed priority on convenient maintenance after a failure. From the perspective of dentists, the water fountain and cuspidor are the places where there is a risk of cross contamination, as they are used by patients and dental hygienists [5,17]. Additionally, in the interviews, it was noted that the water fountain and cuspidor took up great proportions of the external size of the dental unit chair. Thus, the overall satisfaction with the dental unit chair strongly interacted with each of the three factors, and it was highlighted as an important factor.

In previous studies, it has been reported that the dentist’s posture affected the muscle activities in the neck and shoulders in the treatment process [26,27,30], and various components of the dental unit chair may affect the dentist’s posture [6]. This study highlighted the factors that have strong correlations with the overall satisfaction score with each component of the dental unit chair (Table 5). The study results showed that the satisfaction with various components of the dental unit chair differed depending on their design and functional importance (Table 5).

There are some potential limitations of this study. The study included a small number of subjects. Results from more subjects are needed. Thus, it is necessary to conduct an additional study with more subjects, referring to the research methods and results of this in vivo study. In addition, while evaluating the design of the dental unit chair system, the induction of musculoskeletal disorders is an important point to consider. Therefore, the dentist’s knowledge of ergonomic laws is important, and additional studies of musculoskeletal disorders should be performed. In addition, because this study was only from the perspective of dentists, additional studies are needed from the perspective of dental hygienists and patients in the future.

## 5. Conclusions

Within the limitations of this in vivo study, a survey of user satisfaction with the dental unit chair was conducted with the dentists of South Korea, and the factors affecting the satisfaction are as follows:

1. Because the design and appearance quality, maintenance, and overall satisfaction with the water fountain and cuspidor may have the greatest impact on the overall satisfaction with the dental unit chair, the improvement of these factors may bring about the enhancement of the overall satisfaction.
2. Additionally, regarding the dental unit chair components, the following factors have the greatest impact on the overall satisfaction with the dental unit chair: with regard to light, the convenience of color temperature control; with regard to the patient seat, the appropriate length and area for each part; with regard to the foot controller, the ease of operation and pedal pressure during operation; with regard to the water fountain and cuspidor of the dental unit chair, the height and area of the cuspidor and cup position; with regard to the monitor of the dental unit chair, the distance between monitor and operator/patient; with regard to the bracket table and controller of the dental unit chair, the weight; and with regard to the dentist chair, human engineering design. The improvement of these factors may bring about the enhancement of the satisfaction with each component of the dental unit chair.

## Figures and Tables

**Table 1:** User satisfaction questionnaire items.

Components of the Dental Unit Chair	Environmental Factors	Clinical Needs
Light	Convenience of light head movement	Convenience of movement for accurate light irradiation into the patient’s oral cavity and operator’s comfort
	Convenience of color temperature control	Color temperature control in consideration of tooth color discrimination and eye fatigue in various clinical environments
	Influence of illuminance	Illuminance suitable for treatment considering eye fatigue and resin light curing
Patient seat	Appropriate length and area for each part	A seat that has a size suitable for the human body in Korea considering the patient and dentist’s body shape
	Back reclining angle	Optimal backrest reclining angle for patient comfort and dentist posture
	Headrest recline angle	Optimal headrest reclining angle for patient comfort and dentist posture
	Backrest reclining speed	Appropriate speed considering both stability and convenience of operation when laying down or returning the patient to the original position
	Backrest seat thickness	Seat thickness considering the shape of the spine and knee height for the dentist’s correct treatment posture and patient comfort
	Convenience of auxiliary handle	Convenience that can help without interfering with the patient’s movement
Foot controller	Ease of operation	Dentist’s intuitive operation convenience
	Pedal pressure during operation	Precise use of the handpiece and less pressure on the ankle
Water fountain and cuspidor	Ease of operation	Convenience of operation by patients and medical assistants
	The height and area of the cuspidor	Height and area to minimize discomfort during movement
	Cup position	The position of the cup considering the convenience of the patient
	Water purification and wastewater treatment facilities	Pleasant environment and facilities to prevent cross infection
	Water quantity and speed	Adequacy of water supply quantity and water supply speed
	Convenience of cleaning the cuspidor	Automatic cleaning of contaminants and ease of cleaning management
Monitor	Screen size	Visual convenience, various information provision, and monitor size in harmony with the overall medical system
	Distance between monitor and operator/patient	Monitor distance considering visual convenience and movement for dentists and patients
	Convenience to move	Stable and smooth movement
	Resolution	Readability of radiographs, oral photographs, and text
	Arm support	Even after the monitor is moved, it is fixed by supporting the monitor stably without movement
Bracket table and controller	Size	It is a size that can mount various instruments and does not affect the medical environment
	Weight	Weight that is stable and does not burden when moving
	Mounting stability	Stable and convenient mounting of various instruments
	Controller user interface	User interface suitable for the user environment of dentists and medical assistants
	Stability of table arm operation	Stability when moving and fixing the table
Dentist chair	Backrest angle and shape	Backrest angle and shape for dentists’ health and comfort
	Convenience to move	Soft chair movement considering durability and noise
	Human engineering design	Design that harmonizes with the human body and does not burden the dentist, especially with long-term use

**Table 2:** Results of the factor analysis and reliability analysis for the extracted questionnaire items.

Number	Items		Factor Analysis			Cronbach’s Alpha
			Factor Load	Communality	Contribution Rate (%)	
1	Overall evaluation	Price	0.782	0.797	51.504	0.881
2		Versatility of function	0.557	0.929		
3		Design and appearance quality	0.561	0.926		
4		Maintenance	0.682	0.815		
5		Overall satisfaction	0.63	0.9		
6	Light	Convenience of light head movement	0.819	0.878	9.055	0.859
7		Convenience of color temperature control	0.851	0.757		
8		Influence of illuminance	0.829	0.959		
9		Overall satisfaction of light	0.688	0.948		
10	Patient seat	Appropriate length and area for each part	0.634	0.841	6.848	0.898
11		Back reclining angle	0.84	0.832		
12		Head rest recline angle	0.572	0.744		
13		Backrest reclining speed	0.726	0.746		
14		Backrest seat thickness	0.578	0.91		
15		Convenience of auxiliary handle	0.727	0.921		
16		Overall satisfaction of patient seat	0.512	0.853		
17	Foot controller	Ease of operation	0.506	0.936	6.191	0.886
18		Pedal pressure during operation	0.784	0.891		
19		Overall satisfaction of foot controller	0.758	0.896		
20	Water fountain and cuspidor	Ease of operation	0.688	0.934	4.596	0.938
21		The height and area of the cuspidor	0.608	0.937		
22		Cup position	0.596	0.939		
23		Water purification and wastewater treatment facilities	0.794	0.866		
24		Water quantity and speed	0.65	0.853		
25		Convenience of cleaning the cuspidor	0.828	0.942		
26		Overall satisfaction of water fountain and cuspidor	0.759	0.915		
27	Monitor	Screen size	0.845	0.943	3.43	0.963
28		Distance between monitor and operator/patient	0.886	0.892		
29		Convenience to move	0.807	0.89		
30		Resolution	0.57	0.807		
31		Arm support	0.851	0.808		
32		Overall satisfaction of monitor	0.64	0.836		
33	Bracket table and controller	Size	0.839	0.814	3.29	0.974
34		Weight	0.872	0.848		
35		Mounting stability	0.819	0.877		
36		Controller user interface	0.624	0.918		
37		Stability of table arm operation	0.559	0.905		
38		Overall satisfaction of bracket table and controller	0.862	0.779		
39	Dentist chair	Back rest angle and shape	0.789	0.801	2.303	0.93
40		Convenience to move	0.88	0.874		
41		Human engineering design	0.518	0.939		
42		Overall satisfaction of dentist chair	0.717	0.836		

**Table 3:** Study inclusion and exclusion criteria.

Inclusion Criteria	Exclusion Criteria
Clinical experience >3 years. Participants who have sufficient experience in using the dental unit chair system. Participants who fully understand the purpose of this clinical study and can participate actively.	Participants who are inappropriate to participate in clinical trials in the judgment of the clinical trial director because they may affect the clinical trial results. Participants who have no experience in using a dental unit chair system.

**Table 4:** Results of the correlation analysis between the overall satisfaction score of the chair unit and the questionnaire items.

Number	Items		Overall Satisfaction Score	
			Correlation Coefficient	*p*
1	Overall evaluation	Price	0.648	<0.001 *
2		Versatility of function	0.64	<0.001 *
3		Design and appearance quality	0.781	<0.001 *
4		Maintenance	0.784	<0.001 *
5	Light	Convenience of light head movement	0.568	0.002 *
6		Convenience of color temperature control	0.426	0.03 *
7		Influence of illuminance	0.634	0.001 *
8		Overall satisfaction of light	0.629	0.001 *
9	Patient seat	Appropriate length and area for each part	0.403	0.041 *
10		Back reclining angle	0.498	0.01 *
11		Head rest recline angle	0.361	0.07
12		Backrest reclining speed	0.401	0.042 *
13		Backrest seat thickness	0.405	0.04 *
14		Convenience of auxiliary handle	0.504	0.009 *
15		Overall satisfaction of patient seat	0.587	0.002 *
16	Foot controller	Ease of operation	0.679	<0.001 *
17		Pedal pressure during operation	0.645	<0.001 *
18		Overall satisfaction of foot controller	0.581	0.002 *
19	Water fountain and cuspidor	Ease of operation	0.633	0.001 *
20		The height and area of the cuspidor	0.562	0.003 *
21		Cup position	0.609	<0.001 *
22		Water purification and wastewater treatment facilities	0.608	0.001 *
23		Water quantity and speed	0.67	<0.001 *
24		Convenience of cleaning the cuspidor	0.598	0.001 *
25		Overall satisfaction of water fountain and cuspidor	0.703	<0.001 *
26	Monitor	Screen size	0.508	0.008 *
27		Distance between monitor and operator/patient	0.492	0.011 *
28		Convenience to move	0.449	0.021 *
29		Resolution	0.563	0.003 *
30		Arm support	0.555	0.003 *
31		Overall satisfaction of monitor	0.545	0.004 *
32	Bracket table and controller	Size	0.576	0.002 *
33		Weight	0.587	0.002 *
34		Mounting stability	0.627	0.001 *
35		Controller user interface	0.442	0.024 *
36		Stability of table arm operation	0.579	0.002 *
37		Overall satisfaction of bracket table and controller	0.564	0.003 *
38	Dentist chair	Backrest angle and shape	0.45	0.021 *
39		Convenience to move	0.517	0.007 *
40		Human engineering design	0.354	0.076
41		Overall satisfaction of dentist chair	0.369	0.064

* Significance determined using Pearson’s correlation analysis, *p* < 0.05.

**Table 5:** Results of the correlation analysis between the overall satisfaction of each element constituting the chair unit and the questionnaire items.

Number	Items		Overall Satisfaction Score	
			Correlation Coefficient	*p*
1	Light	Convenience of light head movement	0.369	0.064
2		Convenience of color temperature control	0.866	<0.001 *
3		Influence of illuminance	0.803	<0.001 *
4	Patient seat	Appropriate length and area for each part	0.821	<0.001 *
5		Back reclining angle	0.727	<0.001 *
6		Headrest recline angle	0.436	0.026 *
7		Back rest reclining speed	0.700	<0.001 *
8		Back rest seat thickness	0.643	<0.001 *
9		Convenience of auxiliary handle	0.700	<0.001 *
10	Foot controller	Ease of operation	0.736	<0.001 *
11		Pedal pressure during operation	0.737	<0.001 *
12	Water fountain and cuspidor	Ease of operation	0.748	<0.001 *
13		The height and area of the cuspidor	0.824	<0.001 *
14		Cup position	0.862	<0.001 *
15		Water purification and wastewater treatment facilities	0.722	<0.001 *
16		Water quantity and speed	0.686	<0.001 *
17		Convenience of cleaning the cuspidor	0.774	<0.001 *
18	Monitor	Screen size	0.871	<0.001 *
19		Distance between monitor and operator/patient	0.923	<0.001 *
20		Convenience to move	0.877	<0.001 *
21		Resolution	0.811	<0.001 *
22		Arm support	0.865	<0.001 *
23	Bracket table and controller	Size	0.824	<0.001 *
24		Weight	0.948	<0.001 *
25		Mounting stability	0.899	<0.001 *
26		Controller user interface	0.836	<0.001 *
27		Stability of table arm operation	0.899	<0.001 *
28	Dentist chair	Back rest angle and shape	0.768	<0.001 *
29		Convenience to move	0.780	<0.001 *
30		Human engineering design	0.932	<0.001 *

* Significance determined using Pearson’s correlation analysis, *p* < 0.05.

## Data Availability

The data are included within the article.
